# New Research in Obsessive-Compulsive Disorder and Major Depression

**DOI:** 10.3390/brainsci9060140

**Published:** 2019-06-17

**Authors:** Bruno Aouizerate, Emmanuel Haffen

**Affiliations:** 1 Pôle de Psychiatrie Générale et Universitaire, Centre de référence régional des pathologies anxieuses et de la dépression, Centre Expert Dépression Résistante FondaMental, CH Charles Perrens, NutriNeuro, UMR INRA 1286, Université de Bordeaux, 33076 Bordeaux, France; 2 Service de Psychiatrie, CIC-1431 INSERM, CHU de Besançon, laboratoire de Neurosciences, univ. Bourgogne-Franche-Comté, 25030 Créteil, France

Major depression and obsessive-compulsive disorder (OCD) are among the most frequent psychiatric disorders in the general population. They are also frequently associated with one another in daily clinical practice. They generate severe emotional distress and marked impairment in general functioning. They are both characterized by a chronic and/or recurrent course, thereby leading to a profound deterioration of quality of life. Despite significant advances in pharmacological and psychological therapies, 20–30% of patients still respond unsuccessfully to standard medical treatment strategies for these disorders. In this context, one of the major outstanding research challenges is to better identify precise phenotypic profiles through the validation of innovative numerical tools, enabling the online recording and monitoring of the cognitive, emotional, and behavioral components underlying the expression of clinical phenotypes. This is nicely addressed in the article by Briffault et al. [1] from the perspective of opening new “symptoms networks” for the promotion of more homogenous diagnostic categories in psychiatry. In parallel, the development of relevant experimental paradigms is useful for the assessment of disturbances in sensory processes related to the olfactory sphere, which are classically observed in major depression, as part of the core dimensions/symptoms, such as anhedonia, primarily referring to a reduced feeling of pleasure from usually enjoyable activities. This kind of research receives particular attention from Rochet et al. [2] considering both anatomical and functional findings and relying on a translational approach ranging from laboratory animal models to humans for the determination of the brain regions that are more commonly implicated in olfaction, hedonic processes, and major depression. Additionally, there is a large body of literature supporting the implication of biological mechanisms, among which immune function with an abnormal inflammatory response is most often cited. This “inflammatory” theory, which was largely documented in the area of depression, was more recently extended to OCD, as in the article by Lamothe et al. [3] which examines cytokine profiles and white blood cell populations. The authors have also investigated relationships with streptococcal infections and the PANDAS generating a large constellation of neuropsychiatric conditions, especially including OCD that is mainly mediated by disruption in the basal ganglia, which are particularly vulnerable to antineuronal antibodies relying on stimulated autoimmune processes. Beyond the description of these pathophysiological determinants, the articles by Brunelin et al. [4] and Bennabi et al. [5] are respectively dedicated to therapeutic challenges in the utilization of the non-invasive brain stimulation technique tDCS in chronic forms of OCD and major depression that are unresponsive to the conventional medical treatments. Although data are still scarce, tDCS seems to represent a promising alternative for the management of major depression and OCD in substantially reducing the clinical severity with relatively good tolerability. However, these beneficial effects remain to be further confirmed in larger and controlled studies in order to alleviate the methodological concerns that could be raised in the already published trials. To conclude, this Special Issue will therefore provide precious information at the clinical, pathophysiological, and therapeutic levels that could be helpful to a wide readership, ranging from mental health professionals to basic/clinical researchers with a significant interest in increasing their knowledge in the field of major depression and OCD.
